# Differential effects of chronic immunosuppression on behavioral, epigenetic, and Alzheimer’s disease-associated markers in 3xTg-AD mice

**DOI:** 10.1186/s13195-020-00745-9

**Published:** 2021-01-20

**Authors:** Minesh Kapadia, M. Firoz Mian, Donglai Ma, Craig P. Hutton, Amber Azam, Klotilda Narkaj, Chuanhai Cao, Breanna Brown, Bernadeta Michalski, David Morgan, Paul Forsythe, Iva B. Zovkic, Margaret Fahnestock, Boris Sakic

**Affiliations:** 1 Department of Psychiatry and Behavioral Neurosciences, McMaster University, 1280 Main St. West, Hamilton, ON L8S 4K1 Canada; 2 Department of Medicine, McMaster University, Hamilton, ON Canada; 3 Department of Pathology and Molecular Medicine, McMaster University, Hamilton, ON Canada; 4 Department of Psychology, Neuroscience, and Behaviour, McMaster University, Hamilton, ON Canada; 5 Department of Psychology, University of Toronto Mississauga, Mississauga, ON Canada; 6 Department of Pharmaceutical Science, University of South Florida, Tampa, FL USA; 7 Department of Translational Science & Molecular Medicine, Michigan State University, Grand Rapids, MI USA

**Keywords:** Alzheimer’s disease, 3xTg-AD mice, Autoimmunity, Cyclophosphamide, T lymphocytes, Autoantibodies, Amyloid-beta, Tau, BDNF, Histone variants, Sex

## Abstract

**Background:**

Circulating autoantibodies and sex-dependent discrepancy in prevalence are unexplained phenomena of Alzheimer’s disease (AD). Using the 3xTg-AD mouse model, we reported that adult males show early manifestations of systemic autoimmunity, increased emotional reactivity, enhanced expression of the histone variant macroH2A1 in the cerebral cortex, and loss of plaque/tangle pathology. Conversely, adult females display less severe autoimmunity and retain their AD-like phenotype. This study examines the link between immunity and other traits of the current 3xTg-AD model.

**Methods:**

Young 3xTg-AD and wild-type mice drank a sucrose-laced 0.4 mg/ml solution of the immunosuppressant cyclophosphamide on weekends for 5 months. After behavioral phenotyping at 2 and 6 months of age, we assessed organ mass, serologic markers of autoimmunity, molecular markers of early AD pathology, and expression of genes associated with neurodegeneration.

**Results:**

Chronic immunosuppression prevented hematocrit drop and reduced soluble Aβ in 3xTg-AD males while normalizing the expression of histone variant macroH2A1 in 3xTg-AD females. This treatment also reduced hepatosplenomegaly, lowered autoantibody levels, and increased the effector T cell population while decreasing the proportion of regulatory T cells in both sexes. Exposure to cyclophosphamide, however, neither prevented reduced brain mass and BDNF expression nor normalized increased tau and anxiety-related behaviors.

**Conclusion:**

The results suggest that systemic autoimmunity increases soluble Aβ production and affects transcriptional regulation of macroH2A1 in a sex-related manner. Despite the complexity of multisystem interactions, 3xTg-AD mice can be a useful in vivo model for exploring the regulatory role of autoimmunity in the etiology of AD-like neurodegenerative disorders.

## Introduction

Alzheimer’s disease (AD) is a neurodegenerative disorder that disproportionately affects women, both in prevalence and severity [5, 36, 65]. Although the causes for this discrepancy remain poorly understood, factors beyond longevity have been implicated [2, 24]. Recent genomic [44, 47, 64] and clinical studies [8, 62, 77, 104, 111] point to the involvement of the immune system in the etiology of AD. However, the possibility that sex-specific differences in immunity [61] underlie increased disease prevalence in females has not been explored. One limiting factor is the dearth of appropriate animal models in which the causal relationships between the immune system, sex, and AD-like neuropathology can be studied in a controlled and systematic manner.

The triple transgenic (3xTg-AD) mouse model is a widely used tool for studying AD pathogenesis [12] because it develops age-related cognitive impairments and soluble intraneuronal amyloid-beta (Aβ) oligomers by 6 months of age and neuritic plaques and neurofibrillary tangles in the cortex and hippocampus after 12 months of age [13, 82, 83]. The 3xTg-AD mouse model has been instrumental in documenting how Aβ contributes to tauopathy [84, 119], how soluble Aβ and tau contribute to early stages of the disease [43, 93], and how inflammation potentiates neuropathology [54, 60, 114]. More recently, we observed that in 3xTg-AD mice, behavioral deficits similar to mild cognitive impairment appear as a prodrome to subsequent decline in spatial learning/memory task performance [50, 69]. Namely, between 2 and 6 months of age, these mice display pronounced anxiety-related behaviors (e.g., “acrophobia” in the step-down test and elevated plus maze, altered exploration of the open field, and enhanced thigmotaxis in a swimming pool), changes in olfactory sensitivity, and impairments in cognitive flexibility when tested in reversal trials of the Morris water maze [50, 69]. Coinciding with these behavioral changes is the spontaneous development of a progressive systemic autoimmune response, as evidenced by splenomegaly, hepatomegaly, elevated serum levels of anti-nuclear/anti-dsDNA antibodies, low hematocrit, and increased number of double-negative T splenocytes [69]. Importantly, this immune activation in 3xTg-AD males manifests as early as 1.5 month of age, well before the earliest documented signs of neuropathology [69], and persists even at older ages [29]. When compared to wild-type males, AD males also show increased expression of macroH2A1 (mH2A1) [56], which is a variant of the canonical histone H2A, important in neuroplasticity and often upregulated during neurodegenerative processes [31, 49]. Paradoxically, the brains of 1-year-old males no longer show plaque and tangle deposits. This loss of AD-like pathology in some male cohorts was confirmed independently by the donating investigator in 2014 (https://www.jax.org/strain/004807).

In comparison to 3xTg-AD males, 1-year-old 3xTg-AD females still show Aβ plaque deposition in the cortex and hyperphosphorylated tau tangles in the hippocampus [10], in parallel with development of milder autoimmune manifestations [56]. This sex-dependent shift in phenotypic traits is not isolated to a single colony, as several independent groups have documented sex differences in behavior [14, 15, 19, 26, 40, 90, 91, 96, 109, 112, 117], AD-like neuropathology [21, 46, 88, 90, 91], response to environmental enrichment or exercise [3, 38, 42, 116], life span [40, 92], and immunity [40, 42]. Importantly, many of these sex-specific differences are apparent within the first 6 months of life [19, 21, 26, 38, 42, 56, 90, 91, 96, 109]. Jointly, these findings suggest that adult male 3xTg-AD mice develop a stronger autoimmune response than females that alters their behavioral profile early in the course of the disease and is associated with a delay in AD-like pathology in comparison to female littermates. However, no study to date has systematically examined the cause-effect relationship between spontaneous peripheral immune activation, early behavioral dysfunction, and prodromal markers of AD-like pathology in both male and female 3xTg-AD mice. To test the nature of sex-related systemic autoimmunity in the context of the altered 3xTg-AD phenotype, the current study compares molecular, cellular, and functional consequences of prolonged immunosuppressive treatment in adult male and female 3xTg-AD and wild-type mice.

The alkylating agent cyclophosphamide (CY) is effective in arresting systemic autoimmunity via its metabolite phosphoramide mustard, which is formed in cells with low levels of aldehyde dehydrogenase and leads to apoptosis by forming DNA crosslinks. Given that chronic exposure to CY prevents neurodegeneration and normalizes behavior in autoimmune mice [58, 101–103], an identical, well-established protocol that does not involve injection-induced stress was chosen as a treatment modality in the current study. To provide consistency and comparability across studies, previously used behavioral, cellular, and molecular variables [56, 69] were analyzed.

## Materials and methods

### Animals

Colonies of homozygous 3xTg-AD mice containing *PS1*_M146V_, *APP*_swe_, and *tau*_P301L_ mutations and wild-type (WT), non-transgenic controls of the same mixed B6129SF2 background were established from breeders purchased from the Jackson Laboratories (Bar Harbor, ME, USA). In order to age-match the animals, in-house litters born on the same date (± 3 days) were selected for the current study and tested in parallel. Due to inherent differences in the size of litters among 3xTg-AD breeders versus WT breeders, the final number of animals included was 69 3xTg-AD mice and 75 WT mice. Pups were weaned at 21 days of age and housed in same-sex, age-matched groups of 3 to 5 littermates under the following laboratory conditions: 22 °C, 60% humidity, ad lib access to low-fat rodent chow and water in 150 ml leak-proof bottles, and on a reverse 12-h light/dark cycle. They were tail-tattooed with AIMS™ ATS-3 System at the beginning of the study and weighed every 2 weeks. All experimental protocols were performed with the approval of the local Animal Care Committee and the Canadian Council on Animal Care.

### Immunosuppressive treatment

Four-week-old mice were assigned to one of 8 groups (*n* = 13–21 mice/group) according to substrain (3xTg-AD vs. WT), sex (male vs. female), and treatment (CY vs. vehicle, Veh). They were given to drink an aqueous solution of CY (diluted to 0.4 mg/ml, “Procytox,” Baxter, Mississauga, ON), an immunosuppressive drug, laced with 16% sucrose, or sucrose-only solutions (vehicle) in place of water bottles, available ad libitum over the weekends for 5 months. Such a non-invasive administration route was chosen to avoid confounding effects of repeated pain- and restraint-induced stress on behavioral performance [7, 74, 98]. Moreover, due to its metallic taste, CY was laced with sucrose to increase palatability and thus achieve the therapeutic dose range previously shown to attenuate systemic autoimmunity and normalize functional deficits in lupus-prone mice [57]. In order to assess general CY toxicity, we similarly treated WT mice to control for multisystem effects of CY and its metabolites that are not related to the immune system. Mice were individually housed, and either CY or Veh solutions were administered throughout the duration of the study until sacrifice at ~ 6.5 months of age. Total volume consumed per weekend was monitored, and the amount of CY consumed by each mouse was recorded at the beginning of the study (1–2 months of age) and at the end of the study (5–6 months of age). The final number of animals in each group was as follows: WT Veh males (*n* = 16), WT CY males (*n* = 20), 3xTg-AD Veh males (*n* = 13), 3xTg-AD CY males (*n* = 17), WT Veh females (*n* = 18), WT CY females (*n* = 21), 3xTg-AD Veh females (*n* = 14), and 3xTg-AD CY females (*n* = 15).

### Behavioral battery

**Fig. 1:**
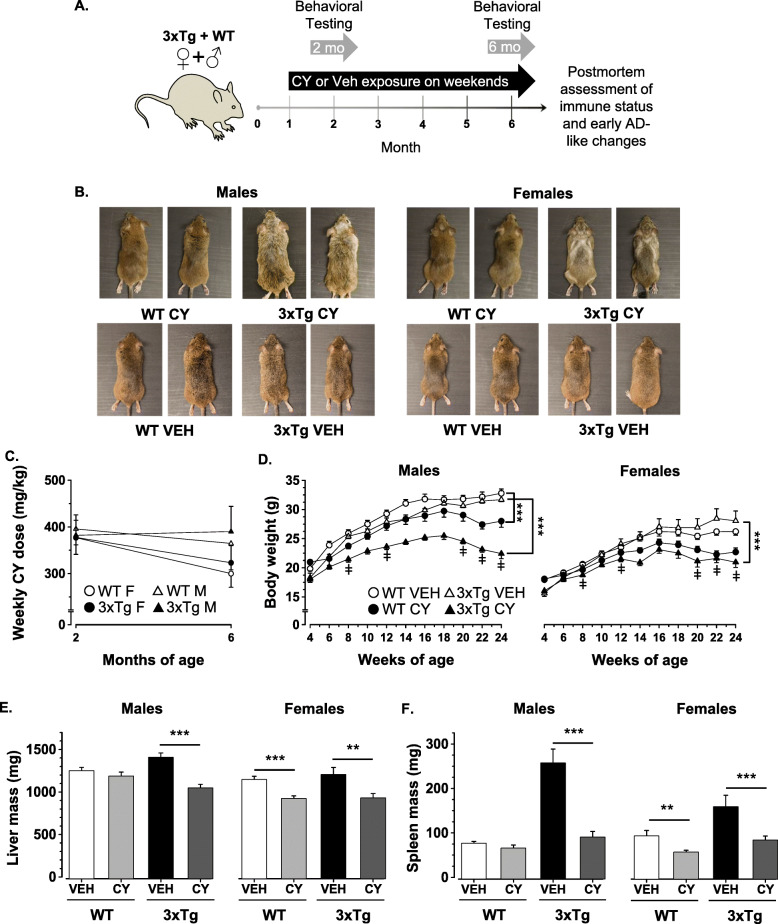
Cyclophosphamide (CY)-induced alterations in peripheral indicators. **a** Study design and experimental timeline diagram depicting the age and testing timepoints for data collected. In brief, 1-month-old 3xTg-AD and WT mice of both sexes were randomly assigned to receive treatment with CY or vehicle solution on weekends until 6.5 months of age (*n* = 13–21 mice/group). All mice were tested in a behavioral battery between 1.5 and 2.5 months of age (2-month timepoint) and re-evaluated between 5.5 and 6.5 months of age (6-month timepoint) to determine the short- and long-term effects of CY treatment. After the behavioral testing was completed, at 6.5 months of age, mice were euthanized and tissues were collected for biomarker assays (see text). **b** Representative photos showing ruffled and depigmented fur in cyclophosphamide (CY)-treated 3xTg-AD males and a distinct, V-pattern of graying commonly seen in 3xTg-AD females. Alterations in fur quality or condition were not noticeable in CY-exposed WT controls or in vehicle-treated (Veh) groups. **c** Mice with access to CY on weekends voluntarily ingested similar doses (mg/kg/weekend) of the immunosuppressant at ~ 2 (WT males, 377 ± 37; 3xTg-AD males, 382 ± 20; WT females, 377 ± 36; 3xTg-AD females, 396 ± 30) and ~ 6 months of age (WT males, 323 ± 37; 3xTg-AD males, 390 ± 54; WT females, 300 ± 30; 3xTg-AD females, 364 ± 56). **d** Sustained exposure to CY (closed symbols), but not vehicle solution (open symbols), induced loss of body mass that was more severe in the 3xTg-AD substrain (triangle) than in WT controls (circles) and became more apparent at older ages (Genotype × Treatment × Week: *F*_1, 120_ = 5.55, *p* < .02). **e** Prolonged treatment with CY abolished substrain differences in liver weight by ameliorating hepatomegaly evident in Veh-treated 6-month-old 3xTg-AD mice (males: *p* < .001; females: *p* < .01). CY also induced more pronounced weight reduction in WT females (*p* < .001), than in WT males. **f** Vehicle-treated 3xTg-AD mice of both sexes exhibited spleen enlargement which was prevented in CY-treated conspecifics (males: *p* < .001; females: *p* < .001). CY treatment in WT females, but not in WT males, also significantly reduced spleen weights in comparison to the Veh group (*p* = .005). WT Veh males (*n* = 16), WT CY males (*n* = 20), 3xTg-AD Veh males (*n* = 13), 3xTg-AD CY males (*n* = 17), WT Veh females (*n* = 18), WT CY females (*n* = 21), 3xTg-AD Veh females (*n* = 13), 3xTg-AD CY females (*n* = 15). Overall group comparisons were carried out using three-way ANOVA (Genotype × Treatment × Sex) followed by post hoc *t* tests. Body weight comparisons were performed using a repeated measures ANOVA with week as a between-subject factor followed by post hoc ANOVAs at each timepoint. Error bars = SEM, *p ≤ .05, **p < .01, ***p < .001, ‡ = Genotype × Treatment interaction

Following an initial 5-day habituation period, all mice underwent behavioral phenotyping from 1.5 to 2.5 months and again from 5.5 to 6.5 months of age (Fig. 1a). These periods correspond to timepoints at which 3xTg-AD mice are documented to exhibit anxiety-like behaviors, altered olfactory sensitivity [50, 69], and learning/memory impairment, accompanied by accumulation of intraneuronal Aβ in the hippocampus and amygdala [13, 82]. In each block, mice were exposed to tests reflective of neurological/sensorimotor function, spontaneous locomotor activity, and emotional reactivity. These tests were performed during the dark phase in the following order: basic reflexes, beam-walking, Rotarod, olfactory sensitivity, T-maze alternation, novel object, open field, step-down, Morris water maze, and spontaneous activity, as described earlier in detail [55, 69, 100].

### Tissue collection

Approximately 7-month-old mice were anesthetized with a ketamine/xylazine cocktail, and retro-orbital blood samples were collected. Whole blood was collected after severing the inferior vena cava and centrifuged for 5 min (10,000*×g*, Eppendorf MiniSpin Plus; Fisher Scientific Canada, Ottawa, ON, Canada). Serum was separated from the clot and stored at − 20 °C for quantification of autoantibodies. Mice were intracardially perfused with ∼ 120 ml of phosphate-buffered saline (PBS) over 5 min, and tissues were harvested and wet weighed as previously described [69]. Spleens were wet weighed, collected in cold PBS, kept on ice, and then processed for flow cytometry analysis of T splenocyte distribution. Brains were wet weighed before separating cortical hemispheres, which were flash frozen in liquid nitrogen and stored at − 80 °C for molecular assays.

### Assessment of autoimmunity markers

Hematocrit was measured to assess the volume percentage of red blood cells in blood, as low hematocrit can be a sign of autoimmune hemolytic anemia. Retro-orbital blood samples from anesthetized animals were collected in heparinized Fisher microhematocrit capillary tubes just prior to sacrifice. Sealed tubes were centrifuged for 10 min in a standard microhematocrit centrifuge (Clay-Adams, Parsippany, NJ, USA) and read in a Critocaps reader.

Anti-nuclear antibody (ANA) positivity in sera, a hallmark of systemic autoimmunity, was assessed using an immunofluorescence assay (HEp2010 cells, EUROIMMUN Canada, Mississauga, ON, Canada) according to the manufacturer’s instructions [56]. Semi-quantitative assessment of nuclear staining patterns was performed by an unbiased assessor according to a 1–4 scale using LED-fluorescence microscopy (EUROStar III, EUROIMMUN). Staining patterns were classified based on standardized nomenclature of ANA-HEp-2 cell patterns established by the International Consensus on Antinuclear Antibody (ANA) Patterns, ICAP [22]. In addition to semi-quantitative scoring of ANA positivity, circulating levels of ANA against double-stranded DNA (dsDNA) in sera were quantified using a fully automated ELISA analyzer (EUROIMMUN Analyzer I) and microtiter plate wells coated with dsDNA complexed with nucleosomes, as described previously [56]. A separate ELISA with microtiter plate wells coated with human Aβ_1–42_ peptide was used to quantify serum anti-Aβ_1–42_ antibody titers, as previously described [80]. Results were expressed as relative optical densities.

Splenocyte single cell suspensions were prepared as described earlier [56] and stained for T cell surface markers: APC-anti-CD3 (T lymphocyte marker, 1:200, BD Biosciences Pharmingen, San Diego, CA, USA), FITC-anti-CD4 (helper T lymphocyte marker, 1:200, eBioscience, San Diego, CA, USA), PE-anti-CD8 (cytotoxic T lymphocyte marker, 1:200, eBioscience), and PE-Cy7-anti-CD25 (Treg marker, 1:300, eBioscience). For Foxp3 intracellular staining, after staining with the above surface markers, cells were fixed and permeabilized using eBioscience Intracellular Foxp3 Transcription kit for 20 min and then stained with PerCP-Cy5.5-anti-Foxp3 antibody (1:200, eBioscience) for 30 min. Data were acquired with BD FACSCanto (Becton Dickinson, Mississauga, ON) and analyzed using FlowJo software (TreeStar, Ashland, OR, USA). Compensation controls were set up with single staining for each of the antibodies, including a negative control, using BD CompBeads (BD Biosciences, San Diego, CA, USA). The gating strategy was based on unstained controls and/or fluorescence-minus-one (FMO) controls. Single and live events were gated based on forward scatter and side scatter plots. For each sample, 100,000 events were acquired, adjusting the forward scatter (FSC) and side scatter (SCC) voltage controls to place the lymphocytes on scale as well as to exclude debris/dead cells.

### Assessment of protein markers

Although the utility of 3xTg-AD mice stems from their age-associated development of both plaque and tangle pathology, emerging data suggests that neuronal dysfunction in AD is triggered by soluble species rather than insoluble tangles and plaques [35, 45]. The cortex was selected as the primary tissue of interest, as it is the site of initial intracellular and extracellular neuropathology in 3xTg-AD mice [46, 82]. Extraction of soluble tau and Aβ species was based on published methods [75, 99]. In brief, cortical samples (approx. 100 mg) were sonicated in tris-buffered saline (TBS) with protease (cOmplete™ ULTRA Tablets, Mini, EASYpack Protease Inhibitor Cocktail) and phosphatase (PhosSTOP EASYpack, phosphatase inhibitor tablets) inhibitors (Roche, Mississauga, ON, Canada) and kept on ice for 5–10 min. For soluble Aβ analysis, TBS homogenates were centrifuged for 20 min at 14,000×*g* at 4 °C and supernatants (S1) were collected, aliquoted, and frozen at − 80 °C until use. For analysis of soluble tau, TBS homogenates were centrifuged for 20 min at 27,000×*g* at 4 °C and supernatants (S1) were collected for subsequent use. To prepare insoluble tau aggregates, S1 pellets were homogenized in salt/sucrose buffer [0.8 M NaCl, 10% sucrose, 10 mM Tris/HCl (pH 7.4), 1 mM ethylene glycol-bis(β-aminoethyl ether)-N′,N′,N′,N′-tetraacetic acid, 1 mM phenylmethylsulfonyl fluoride] and centrifuged for 20 min at 27,000×*g* at 4 °C. The resultant supernatant (S2) was adjusted to 1% sarkosyl, incubated for 1 h at 37 °C, and centrifuged at 150,000×*g* for 1 h at 4 °C. The sarkosyl-insoluble pellet was then re-suspended in TE buffer [10 mM Tris/HCl (pH 8.0), 1 mM ethylene diamine tetraacetic acid] and stored at − 80 °C for subsequent analysis. Protein concentrations in each fraction were measured using a detergent-compatible protein assay (Bio-Rad Laboratories, Mississauga, ON, Canada).

Aβ_42_ protein levels in TBS-soluble S1 fractions were measured by Chemiluminescent BetaMark x-42 ELISA per the manufacturer’s instructions (BioLegend, San Diego, CA, USA). Concentrations were acquired with a MultiskanGO and SkanIt software (Thermo Scientific, Nepean, ON, Canada) at 620 nm. This ELISA recognizes both mouse and human Aβ_42_, and therefore, mouse Aβ_42_ values assayed in the corresponding wild-type groups were subtracted as background. Values are presented as pg human Aβ_42_ per mg of total protein.

TBS-soluble and sarkosyl-insoluble total tau and phosphorylated tau were measured using western blotting. Ten and 15 μg of total protein were resolved on 10% gels and transferred to polyvinylidene fluoride membranes (Bio-Rad, Hercules, CA, USA) for analysis of soluble and insoluble tau species, respectively. The membranes were treated as described [75] for detection with primary antibodies anti-tau (tau46, 1:1000; Covance, Princeton, NJ, USA) and anti-phospho-tau (D9F4G, 1:1000; Cell Signaling Technology, Danvers, MA, USA), which recognize both mouse and human tau [89] and incubated in secondary antibodies IRDye 680-conjugated goat anti-rabbit and IRDye 800CW-conjugated goat anti-mouse (1:10,000, Li-Cor Biosciences, Lincoln, NE, USA). Band intensities were quantified by densitometry by normalizing to mouse β-actin monoclonal antibody (1:10,000; BioLegend).

### Assessment of RNA expression

RNA was extracted from cortical samples in TRIzol using RNeasy spin columns (Qiagen, Mississauga, ON), complementary DNA was synthesized, and quantitative real-time polymerase chain reaction was performed as described previously [75]. Primers were designed using Primer3 software (http://bioinfo.ut.ee/primer3/) and ordered from IDT (Coralville, IA, USA). BDNF mRNA copy number in each sample was normalized to its β-actin mRNA copy number [56, 97]. The macroH2A variant of the canonical histone H2A is encoded by two genes that produce distinct proteins, *H2afy* (encodes mH2A1) and *H2afy2* (encodes mH2A2). Expression of both was analyzed as described previously [123]. Genes of interest were normalized against the geometric mean of GAPDH and HPRT, and relative enrichment was normalized to vehicle-treated WT controls. All primer sequences are shown in Table 1.

**Table 1:** Primer sequences used for qRT-PCR

***Gene***	***Accession***	***Forward primer***	***Reverse primer***
*BDNF*	NM_001048139.1	GCGGCAGATAAAAAGACTGC	CTTATGAATCGCCAGCCAAT
*β-actin*	NM_007393.5	AGCCATGTACGTAGCCATCC	CTCTCAGCTGTGGTGGTGAA
*H2afy*	NM_001159513.1	CCCGGAAGTCTAAGAAGCAGGG	AGGATTGATTATGGCCTCCACC
*H2afy2*	NM_207000.2	CGTTCCCCAGTGGCAGAAACT	CCTGCACGTAGATGCCGAT
*Gapdh*	NM_001289726.1	GTGGAGTCATACTGGAACATGTAG	AATGGTGAAGGTCGGTGTG
*Hprt*	NM_013556.2	GGAGTCCTGTTGATGTTGCCAGTA	GGGACGCAGCAACTGACATTTCTA

### Statistical analysis

We previously described Genotype [69] and Sex-related differences in 3xTg-AD mice [56]. However, the focus of this study was significant Genotype × Treatment × Sex or Genotype × Treatment interactions. Raw data analyses were performed using SPSS 20 software (IBM Corp., Armonk, NY, USA). Normal distribution of the data was tested by the Shapiro-Wilk test. When data departed from normality, the overall assumption was that parametric tests were robust enough to detect significant group differences, since the cohorts were independent and population variances were comparable, as revealed by Levene’s test. Analysis of variance (ANOVA), ANOVA with repeated measures, analysis of covariance (ANCOVA), and chi-square test were used for group comparisons. Treatment, Genotype, and Sex were considered between-group factors, and Age or Week as within-group factors, where applicable. If significant interactions were detected, Student’s *t* test was used in post hoc comparisons. Partial eta-squared (η2p) and generalized eta-squared (η2g) were used as measures of effect size for all effects and interactions reported for ANOVAs [95] and ANOVA with repeated measures [6], respectively. For reference, Cohen’s benchmarks for small (0.01), medium (0.06), and large (0.14) effects are recommended for these measures [27, 37, 95]. Pearson’s correlation coefficients were calculated when examining bivariate linear relationships for normal variables. The criterion for statistical significance was set at *p* ≤ .05. Graphs display mean values ± SEM. Significant differences of *p* ≤ .05, *p* < .01, and *p* < .001 are shown as *, **, and ***, respectively.

## Results

### Peripheral effects

The earliest observable effects of sustained CY intake were the development of distinct patterns of fur graying in 3xTg-AD mice, noticeable after the second month of exposure. Representative photos exemplify commonly observed ruffled and gray hair in 3xTg-AD males at 6 months of age (Fig. 1b). In contrast, affected age-matched 3xTg-AD females exhibited a symmetrical, V-like pattern of discoloration. These effects were not seen in CY-treated WT controls or in 3xTg-AD mice exposed to vehicle solution. They were not associated with differences in CY dosage, as drug-treated 3xTg-AD and WT groups ingested comparable amounts of CY when individual intake was measured over single weekends at ~ 2 months (Genotype: *F*_1, 69_ = 1.098, n.s., η2p = .02; Sex: *F*_1, 69_ = 1.20, n.s., η2p = .02) and ~ 6 months of age (Genotype: *F*_1, 67_ = 2.287, n.s., η2p = .03; Sex: *F*_1, 67_ = .314, n.s., η2*p* = .01, Fig. 1c). Despite this similarity, CY-treated 3xTg-AD mice showed more profound weight loss than CY-treated WT controls, which became more apparent with time (Genotype × Treatment × Week: *F*_9, 1080_ = 3.280, *p* < .001, η2g = .03, Fig. 1d).

Given a positive correlation between body and liver weight at sacrifice (*r*_128_ = 0.797, *p* < .001), body weight was used as a covariate in ANCOVA, which revealed heavier livers in 3xTg-AD mice than in WT controls (Genotype: *F*_1, 119_ = 10.840, *p* < .001, η2p = .083). Sustained exposure to CY reduced liver weight comparably in all groups except in WT male mice (Genotype × Treatment × Sex: *F*_1, 119_ = 5.207, *p* = .024, η2p = .042, Fig. 1e). Although exposure to CY reduced spleen weight in a similar pattern (Treatment: *F*_1, 119_ = 24.743, *p* < .001, η2p = .172, Fig. 1f), this effect was most profound in 3xTg-AD males (Genotype × Treatment × Sex: *F*_1, 120_ = 8.259, *p* = .005, η2p = .065).

### Splenic T lymphocytes

**Fig. 2:**
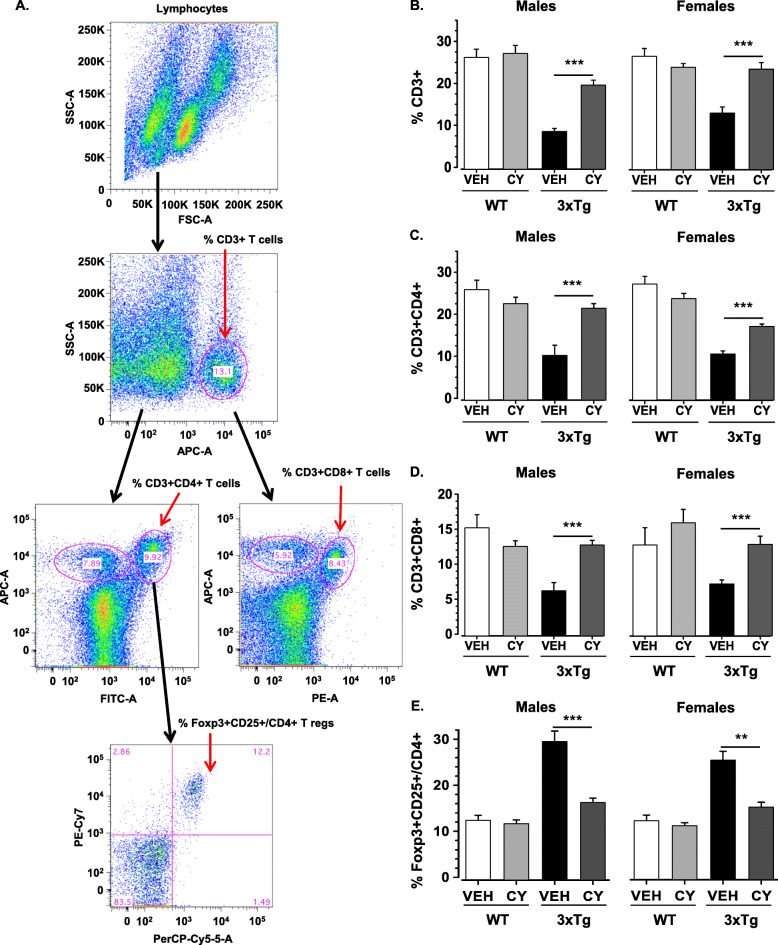
Effects of generalized immunosuppression on T splenocyte populations. **a** The figure illustrates the gating strategies employed to identify the distribution of T lymphocytes in the spleen. Debris was excluded, and lymphocytes included, using a forward scatter area (FSC-A) versus side scatter area (SSC-A) gate. Single cells (singlets) were then selected on a FSC-A versus FSC-W plot. This population was then analyzed in a FL1/SSC plot in order to quantify the percentage of CD3-APC positive cells. An FL2/FL1 plot was used to quantify the percentage of CD4-FITC and CD3-APC positive cells in the total live cell population. Similarly, an FL3/FL1 plot was used to quantify the percentage of CD8-PE and CD3-APC positive cells in the total live cell population. To determine Foxp3+CD25+ Tregs, CD3+CD4+ T cells were further gated on the SSC (FL2) for CD25-PE-Cy7 and FSC (FL1) for Foxp3-PerCP-Cy5.5. The cell population at Q2 are the % Foxp3+CD25+ /CD4+ Tregs. For each sample, 100,000 events were acquired. **b** Analysis of splenic T lymphocytes revealed that 3xTg-AD males treated with CY exhibited a significant increase in spleen-derived CD3^+^ T cells compared to vehicle-treated littermates (*p* < .001). This effect occurred independent of sex, as CY also normalized low CD3^+^ T cells in vehicle-treated 3xTg-AD females (*p* < .001). The loss of CD3^+^ T cells was more pronounced in 3xTg-AD males than females (*p* < .001), while a similar sex difference was not seen in WT controls. **c** CY partially restored low CD3^+^CD4^+^ T cell counts apparent in vehicle-treated 3xTg-AD males (*p* < .001) and females (*p* < .001). **d** CY also normalized low CD3^+^CD8^+^ T cells in the 3xTg-AD substrain (*p* < .001) such that levels were comparable to WT controls. **e** In addition to mitigating the loss of T effector cells, CY treatment abated the rise in the proportion of Foxp3+CD25+/CD4+ Tregs apparent in vehicle-treated 3xTg-AD males (*p* < .001) and females (*p* < .001). WT Veh males (*n* = 5–8), WT CY males (*n* = 5–11), 3xTg-AD Veh males (*n* = 5–7), 3xTg-AD CY males (*n* = 5–10), WT Veh females (*n* = 5–8), WT CY females (*n* = 5–10), 3xTg-AD Veh females (*n* = 5–6), 3xTg-AD CY females (*n* = 5–6). Overall group comparisons were carried out using three-way ANOVA (Genotype × Treatment × Sex) followed by post hoc *t* tests. Error bars = SEM, *p ≤ .05, **p < .01, ***p < .001

The loss of CD4/CD8 markers and the emergence of “double-negative” clones of T cells are well-established phenomena in systemic autoimmunity [113, 115]. Considering that the spleen is a major source of immune cells [71], we investigated if CY alters the splenic distribution of T cell populations using flow cytometry. The strategies employed to gate CD3^+^, CD3^+^CD4^+^, CD3^+^CD8^+^, and Foxp3^+^CD25^+^CD4^+^ cells are shown in Fig. 2a. Chronic intake of CY mitigated the loss of CD3^+^ cells in 3xTg-AD mice, irrespective of sex and without affecting WT controls (Genotype × Treatment: *F*_1, 58_ = 25.809, *p* < .001, η2p = .31, Fig. 2b). Compared to age-matched WT groups (which did not show sex differences), 3xTg-AD males had fewer CD3^+^ cells in comparison to their female conspecifics (Genotype × Sex: *F*_1, 58_ = 5.994, *p* = .017, η2p = .09). Importantly, CY similarly prevented the decline of CD3^+^CD4^+^ T cells (Genotype × Treatment: *F*_1, 58_ = 27.923, *p* < .001, η2p = .33, Fig. 2c) and CD3^+^CD8^+^ T cells in 3xTg-AD mice (Genotype × Treatment: *F*_1, 58_ = 7.136, *p* = .01, η2p = .11, Fig. 2d). We observed that the proportion of CD4+ regulatory T cells (Tregs) expressing CD25 and Foxp3 was higher in both male and female 3xTg-AD mice compared to WT conspecifics (Genotype: *F*_1, 32_ = 101.511, *p* < .001, η2p = .76, Fig. 2e). Again, chronic intake of CY attenuated the shift in balance towards T regulatory cells in the CD4+ population in 3xTg-AD mice, irrespective of sex and without affecting WT controls (Genotype × Treatment: *F*_1, 32_ = 31.464, *p* < .001, η2p = .49, Fig. 2e).

In our original report [69], we made an attempt to compare lymphocyte populations in the bone marrow (which is a primary lymphoid organ) by flushing cells from the medullary cavity of femoral bones dissected from 1-year-old males. We were unable to do this comparison because a needle could not be inserted into the femoral cavity in 3xTg-AD males due to ossification. Furthermore, the femur was solid and pale, suggesting an absence of bone marrow cells (data not reported). Interestingly, in comparison to other groups, sustained CY treatment restored normal, red appearance of the femur of 6-month-old 3xTg-AD males (supplemental data).

### Serological measures

**Fig. 3:**
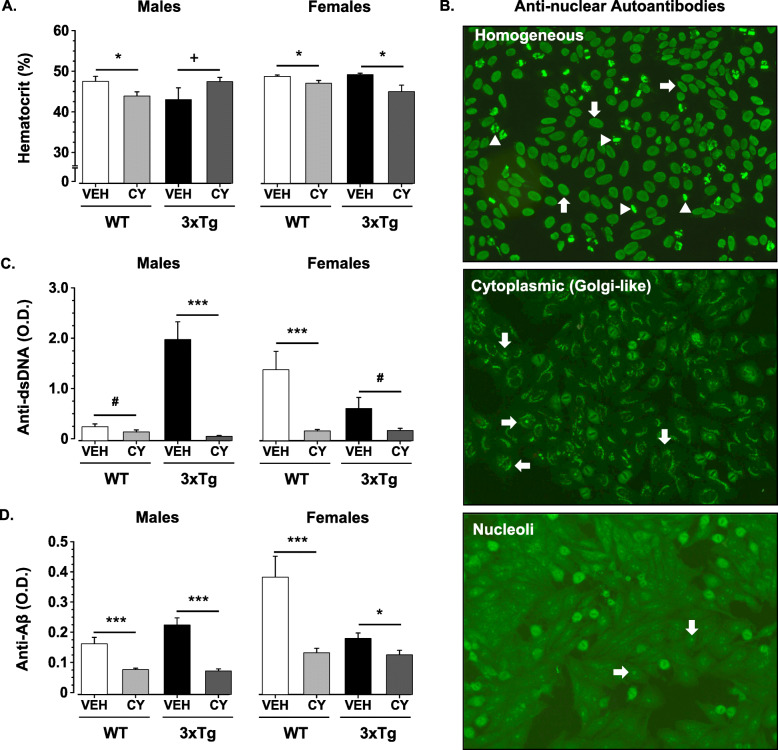
Effects of generalized immunosuppression on serological markers of autoimmunity. **a** Immunosuppression reduced hematocrit in comparison to vehicle-treated mice in all groups except 3xTg-AD males (WT males: *p* = .039; WT females: *p* = .035; 3xTg-AD females: *p* = .02). **b** CY-treated 3xTg-AD and WT mice were negative for serum anti-nuclear antibodies (ANA), but distinct staining patterns were noted in Veh-treated animals. Most common was homogeneous staining of the cell nucleus (white arrows in top panel) and chromosomes (white arrowheads in top panel). Serum from some animals displayed distinct cytoplasmic (Golgi-like) staining (white arrows in middle panel) while others showed a nucleoli pattern (white arrows in lower panel). **c** CY exposure reduced anti-double-stranded DNA (anti-dsDNA) in the sera of all groups (Treatment: *F*_1, 120_ = 28.951, *p* < .001) and, in particular, in 3xTg-AD males (*p* < .001 vs. vehicle-treated 3xTg-AD males) and WT females (*p* < .001 vs. vehicle-treated WT females). **d** Although CY treatment reduced serum anti-Aβ_42_ antibodies in all groups, the antibodies were elevated most strongly in the vehicle-treated WT female group. WT Veh males (*n* = 8–16), WT CY males (*n* = 11–20), 3xTg-AD Veh males (*n* = 7–13), 3xTg-AD CY males (*n* = 10–17), WT Veh females (*n* = 8–18), WT CY females (*n* = 10–21), 3xTg-AD Veh females (*n* = 6–13), 3xTg-AD CY females (*n* = 7–15). Overall group comparisons were carried out using three-way ANOVA (Genotype × Treatment × Sex) followed by post hoc *t* tests. Error bars = SEM, *p ≤ .05, **p < .01, ***p < .001, # = overall treatment effect, + = Genotype × Treatment × Sex interaction

By 6 months of age, 3xTg-AD males (and females to a lesser degree) exhibit robust signs of autoimmunity including low hematocrit and hyperproduction of serum autoantibodies to nuclear antigens [56]. In comparison to Veh controls, prolonged CY exposure lowered hematocrit in all groups except 3xTg-AD males (Genotype × Treatment × Sex: *F*_1, 73_ = 8.399, *p* = .005, η2p = .10, Fig. 3a). This genotype- and sex-dependent effect of CY was accompanied by pronounced alterations in serum autoantibodies to nuclear antigens (ANA; *χ*^2^ = 60.596, df = 7, *p* < .001, Table 2). Although weak ANA reactivity was noted in 3 out of a total 68 (3/68) CY-treated mice, serum samples from ~ 60% of Veh-treated animals showed distinct staining patterns dependent on genotype and sex (Fig. 3b). In particular, a subset of WT control males (3/16) displayed cytoplasmic (Golgi-like) staining, while nearly all 3xTg-AD males (12/13) exhibited moderate to strong homogeneous staining of the nucleus and chromosomes. Interestingly, serum samples from two thirds of Veh WT females also produced staining of the nucleus (9/18) and nucleoli (3/18). In line with these qualitative findings, exposure to CY reduced serum levels of antibodies to dsDNA in all treated groups (Treatment: *F*_1, 119_ = 45.126, *p* < .001, η2p = .28, Fig. 3c). However, this mitigation was more prominent in 3xTg-AD males, which exhibited higher levels of anti-dsDNA than 3xTg-AD females or WT female controls (Genotype × Treatment × Sex: *F*_1, 119_ = 22.256, *p* < .001, η2p = .16). Consistent with these effects, CY also reduced Aβ antibody titers in all groups (Treatment: *F*_1, 120_ = 36.358, *p* < .001, η2p = .23, Fig. 3d). Interestingly, this reduction was more pronounced in WT females, where the Veh group showed the highest levels of Aβ autoantibodies (Genotype × Treatment × Sex: *F*_1, 119_ = 8.454, *p* = .004, η2p = .07, Fig. 3d).

**Table 2:** Frequencies of serum autoantibodies to nuclear antigens (ANA)

***Group***	***ANA positivity rate***	***Homogeneous ANA pattern***	***Cytoplasmic (Golgi-like) ANA pattern***	***Nucleoli ANA pattern***
*WT male VEH*	5/16 = 31.3%	2/16 = 12.5%	3/16 = 18.6%	
*WT male CY*	1/20 = 5%	1/20 = 5%		
*3xTg male VEH*	12/13 = 92.3%	11/13 = 84.6%	1/13 = 7.7%	
*3xTg male CY*	0/17 = 0%			
*WT female VEH*	12/18 = 66.7%	9/18 = 50%		3/18 = 16.7%
*WT female CY*	1/21 = 4.8%	1/21 = 4.8%		
*3xTg female VEH*	4/14 = 28.6%	3/14 = 21.4%		1/14 = 7.1%
*3xTg female CY*	1/15 = 6.7%	1/15 = 6.7%		

### Behavior

**Fig. 4:**
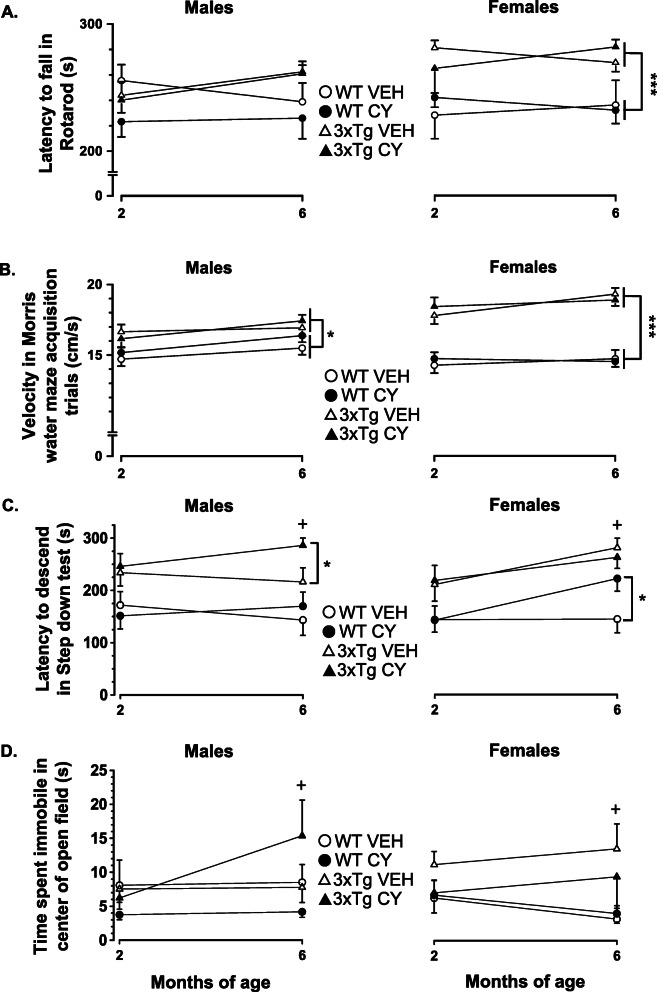
Cyclophosphamide (CY) modulation of anxiety-like behaviors in 3xTg-AD mice at 2 and 6 months of age. **a** Performance in the Rotarod remained superior for 3xTg-AD mice (triangles), irrespective of sex, in comparison to age-matched WT controls (circles) (Genotype: *F*_1, 123_ = 11.334, *p* = .001, η2p = .083). Acute or prolonged CY treatment (closed symbols) did not significantly alter the latency to fall off the Rotarod. **b** Two- and 6-month-old 3xTg-AD males and females swam faster than age-matched WT controls in the Morris water maze acquisition trials (Genotype: *F*_1, 121_ = 72.442, *p* < .001, η2p = .374). CY did not alter the swimming speed of 3xTg-AD mice or WT controls. **c** From an early age, 3xTg-AD males and females took longer than sex-matched WT controls to descend from an elevated platform in the step-down test, consistent with “acrophobia” (Genotype: *F*_1, 126_ = 15.876, *p* < .001). After several months of CY exposure, 3xTg-AD males, but not WT controls, took longer to complete the step-down test in comparison to vehicle-treated animals, suggesting that prolonged immunosuppression exacerbated anxiety-like behavior (Genotype × Treatment × Sex: *F*_1, 124_ = 3.921, *p* = .05). Sustained CY intake had no discernable impact on the step-down performance of 3xTg-AD females, but WT controls (similar to 3xTg-AD males) took longer to complete the task at 6 months of age. **d** In the open field test, CY-treated 3xTg-AD males spent the most time immobile in the center of a large open field (Genotype × Treatment × Sex: *F*_1, 123_ = 4.092, *p* = .045). WT Veh males (*n* = 16), WT CY males (*n* = 20), 3xTg-AD Veh males (*n* = 13), 3xTg-AD CY males (*n* = 17), WT Veh females (*n* = 18), WT CY females (*n* = 21), 3xTg-AD Veh females (*n* = 13), 3xTg-AD CY females (*n* = 15). Overall group comparisons were carried out using three-way ANOVA (Genotype × Treatment × Sex) followed by post hoc *t* tests. Error bars = SEM, *p ≤ .05, **p < .01, ***p < .001, # = overall treatment effect, + = Genotype × Treatment × Sex interaction

Consistent with our previous study [69], data collected with a large behavioral battery demonstrated significant genotype differences in tests of motor coordination/strength, spontaneous activities, and performance in spatial learning/memory tasks (Table 3). We also documented significant Genotype by Sex interactions in the beam-walking test, spontaneous activities, and reversal learning trials in the Morris water maze. When compared to WT groups, 3xTg-AD females performed better in the beam-walking task of visuomotor coordination (as measured by shorter traversing time) and in the basket test (reflecting muscle strength). 3xTg-AD mice, irrespective of sex, exhibited superior performance in the Rotarod test for balance and endurance, as measured by longer latency to fall compared to WT mice (Genotype: *F*_1, 123_ = 11.334, *p* = .001, η2p = .083, Fig. 4a). When tested in the Morris water maze for spatial learning/memory assessment, male and female 3xTg-AD mice swam quicker than age-matched WT controls (Genotype: *F*_1, 121_ = 72.442, *p* < .001, η2p = .374, Fig. 4b). The superior performance of 3xTg-AD mice in these measures of basic sensorimotor evaluation rules out general deficits in locomotion or exploration in this strain. Moreover, 3xTg-AD mice did not show robust deficits in Morris water maze acquisition trials in comparison to WT controls, but 3xTg-AD females performed poorer than 3xTg-AD males in reversal acquisition trials. CY increased water consumption (Treatment: *F*_1, 110_ = 5.136, *p* = .025, η2p = .045; Table 3) and food intake (Treatment: *F*_1, 113_ = 5.377, *p* = .022, η2p = .045; Table 3) in both males and females, irrespective of their genotype and testing age, suggesting that the more robust reductions in body and liver weights in 3xTg-AD mice are not due to reduced caloric or water intake. Importantly, exposure to CY did not have a significant effect on simple reflexes, olfactory sensitivity, T-maze alternation rate, spontaneous activity, or water maze performance, either at 2 or 6 months of age. These results jointly suggest that basic neurological function, muscle strength, motor coordination, spontaneous locomotion, and learning/memory capacity were not significantly altered by chronic CY exposure. Our analysis therefore highlights tests which showed significant effects of CY on genotype and/or sex differences.

**Table 3:** Summary of behavioral data collected. Descriptions of methodology and variables tested are described in detail in previous reports [55, 69, 100]

***Behavioral domain***	***Test***	***Measure***	***Significant test of between-subject factor(s)***	***Alterations in 3xTg-AD vs. WT***	***Effect of immunosuppression***
Sensorimotor coordination, strength	*Beam-walking*	Latency to traverse beam	Genotype × Sex: *F*_1, 122_ = 6.889, *p* = .010, η2p = .053	↓ in females	Null
		Beam slips	Genotype × Sex: *F*_1, 122_ = 5.867, *p* = .017, η2p = .046	↓ in females	Null
	*Rotarod*	Latency to fall	Genotype: *F*_1, 123_ = 11.334, *p* = .001, η2p = .083	↑ in both sexes	Null
	*Basket test*	Latency to fall	Genotype × Sex: *F*_1, 72_ = 7.449, *p* = .008, η2p = .094	↑ in females	Null
Sensory	*Olfactory sensitivity to peanut butter (PB)*	Sniffing duration—0.01% PB	Not significant	None	Null
		Sniffing duration—0.1% PB	Sex: *F*_1, 123_ = 7.356, *p* = .008, η2p = .056	None	Null
		Sniffing duration—1% PB	Not significant	None	Null
Anxiety-like behavior	*Step down*	Latency to descend	Genotype: *F*_1, 122_ = 38.334, *p* < .001, η2p = .236	↑ in both sexes	**↑ in 3xTg-AD males and WT females**
			6 months: Genotype × Treatment × Sex: *F*_1, 124_ = 3.921, *p* = .05, η2p = .03	↑ in both sexes	
	*Open field*	Fecal boli	Genotype: *F*_1, 122_ = 23.332, *p* < .001, η2p = .16	↑ in both sexes	Null
		Distance moved	Genotype: *F*_1, 123_ = 13.623, *p* < .001, η2p = .1	↓ in both sexes	Null
		Frequency of visits to center	Not significant	None	Null
		Latency to enter center	Not significant	None	Null
		Distance moved in center	Not significant	None	Null
		Time spent moving in center	Genotype × Treatment × Sex: *F*_1, 122_ = 3.970, *p* = .049, η2p = .043	↑ in females	**↑ in 3xTg-AD males and ↓ in 3xTg-AD females**
		Time spent immobile in center	Genotype × Treatment × Sex: *F*_1, 122_ = 4.180, *p* = .043, η2p = .033	↑ in both sexes (only at 6 months)	**↑ in 3xTg-AD males**
		Thigmotaxis duration	Genotype: *F*_1, 123_ = 4.525, *p* = .035, η2p = .035	↓ in both sexes (males only at 6 months)	Null
		Velocity	Genotype: *F*_1, 123_ = 5.292, *p* = .023, η2p = .041	↓ in both sexes (females only at 6 months)	↓ in both sexes and strains
			Treatment: *F*_1, 123_ = 5.062, *p* = .026, η2p = .04		
	*Novel object*	Object contact duration	Not significant	None	Null
		Object contact frequency	Not significant	None	Null
		Object contact latency	Not significant	None	Null
Spontaneous behaviors	*Automated activity boxes (INBEST)*	Water intake	Treatment: *F*_1, 110_ = 5.136, *p* = .025, η2p = .045	None	↑ in both sexes and strains
		Sucrose (4%) intake	Treatment: *F*_1, 109_ = 24.437, *p* < .001, η2p = .183	↓ in females	↓ in both sexes and strains
			Genotype × Sex: *F*_1, 109_ = 9.970, *p* = .002, η2p = .084		
		Food intake	Treatment: *F*_1, 113_ = 5.377, *p* = .022, η2p = .045	↑ in males (only at 6 months)	↑ in both sexes and strains
			Genotype × Sex: *F*_1, 113_ = 6.406, *p* = .013, η2p = .054		
		Running wheel rotations	Not significant	None	Null
Working memory	*T-maze*	Spontaneous alternation rate	6 months: Treatment × Sex: *F*_1, 131_ = 4.472, η2p = .036	None	↓ in males and ↑ in females
Spatial learning and memory	Morris water maze	Cue trials—path distance	Not significant	None	Null
		Cue trials—latency	Genotype: *F*_1, 122_ = 8.472, *p* = .004, η2p = .065	↓ in both sexes	Null
		Cue trials—velocity	Genotype: *F*_1, 122_ = 26.806, *p* < .001, η2p = .18	↑ in both sexes	Null
		Acquisition trials—path distance	Genotype × Timepoint × Day: *F*_3, 363_ = 3.502, *p* = .016, η2p = .028	↑ in both sexes (on day 1, 2 months)	Null
		Acquisition trials—latency	Not significant	None	Null
		Acquisition trials—velocity	Genotype: *F*_1, 121_ = 72.442, *p* < .001, η2p = .374	↑ in both sexes	Null
		Probe trials—time spent in target quadrant	Not significant	None	Null
		Reversal cue trials—path distance	Sex: *F*_1, 121_ = 7.704, *p* = .006, η2p = .06	None	Null
		Reversal cue trials—latency	Genotype: *F*_1, 121_ = 10.814, *p* < .001, η2p = .082	↓ in both sexes	Null
		Reversal cue trials—velocity	Genotype: *F*_1, 122_ = 28.250, *p* < .001, η2p = .189	↑ in both sexes	Null
		Reversal acquisition trials—path distance	Genotype × Sex: *F*_1, 121_ = 6.871, *p* = .01, η2p = .054	↑ in females	Null
		Reversal acquisition trials—latency	Sex: *F*_1, 121_ = 10.416, *p* = .002, η2p = .079	None	Null
		Reversal acquisition trials—velocity	Genotype × Sex: *F*_1, 121_ = 22.711, *p* < .001, η2p = .158	↑ in females	Null
		Reversal acquisition trials—time spent in previous quadrant	Genotype × Sex: *F*_1, 121_ = 6.114, *p* = .015, η2p = .048	↑ in females	Null

Two-month-old 3xTg-AD mice showed a longer latency to step down from an elevated platform than WT controls (Genotype: *F*_1, 126_ = 15.876, *p* < .001, η2p = .11, Fig. 4c). When re-tested at an older age, CY-treated 3xTg-AD males and WT females were slower to descend than respective Veh-treated controls (Genotype × Treatment × Sex: *F*_1, 124_ = 3.921, *p* = .05, η2p = .03).

In the open field test, 3xTg-AD mice defecated more (Genotype: *F*_1, 122_ = 23.332, *p* < .001, η2g = .160) and traveled less than WT controls (Genotype: *F*_1, 123_ = 13.623, *p* < .001, η2g = .10; data not shown). Although exposure to CY failed to abolish these differences, it increased time spent in the center of the arena by 3xTg-AD mice in a sex-specific manner (Genotype × Treatment × Sex: *F*_1, 124_ = 4.178, *p* = .043, η2g = .03). Namely, prolonged exposure to CY increased center duration in 3xTg-AD males but reduced it in 3xTg-AD females at 6 months (Genotype × Treatment × Sex: *F*_1, 123_ = 5.752, *p* = .018, η2p = .05, data not shown). The time spent in the center of the open field correlated significantly with immobility time (2 months: *r*_134_ = .834, *p* < .001; 6 months: *r*_130_ = .874, *p* < .001). Video-tracking analysis revealed genotype- and sex-specific effects in CY groups (Genotype × Treatment × Sex: *F*_1, 122_ = 4.18, *p* = .043, η2g = .03). Namely, immobility time was increased significantly in CY-treated 3xTg-AD males when tested at 6 months of age (Genotype × Treatment × Sex: *F*_1, 122_ = 4.092, *p* = .045, η2p = .03, Fig. 4d).

### Neuropathology

**Fig. 5:**
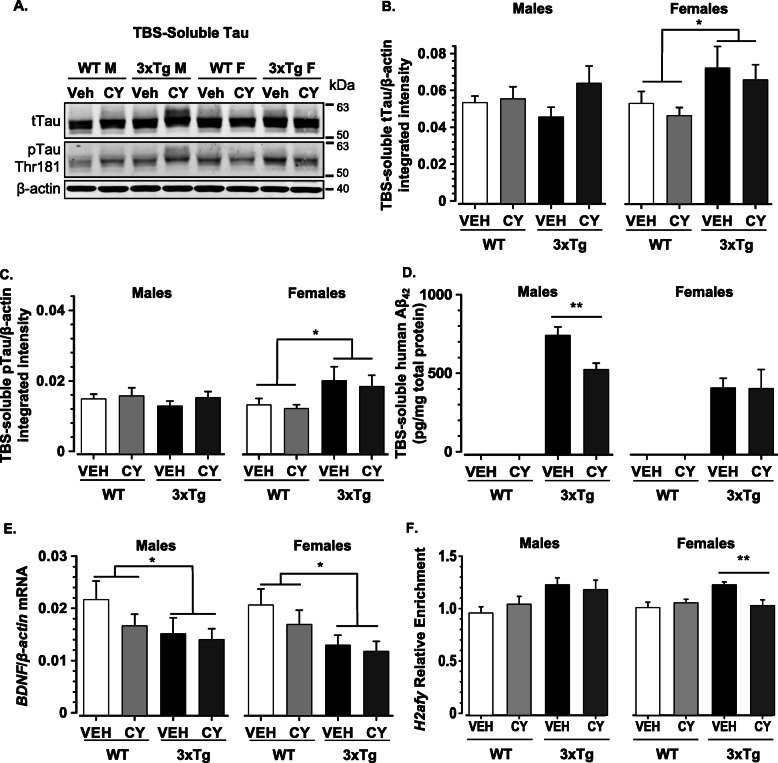
Effects of generalized immunosuppression on soluble and epigenetic markers of neurodegeneration*.* **a** Representative western blot illustrating total tau, phosphorylated tau, and β-actin protein levels in TBS homogenates extracted from the cortex of 6-month-old mice. **b** Densitometric analysis of western blots revealed that 6-month-old 3xTg-AD females had elevated levels of TBS-soluble total tau (normalized to β-actin) in comparison to WT controls, but this genotype-related difference was not noted in males (Genotype × Sex: *F*_1, 81_ = 18.77, *p* < .001) and was not altered by CY immunosuppression. **c** 3xTg-AD females also exhibited an increase in phospho-tau (Thr181) in comparison to all other groups (Genotype × Sex: *F*_1, 81_ = 16.354, *p* < .001), but CY had no appreciable effect on the phosphorylation status of tau. **d** Soluble human Aβ_42_ was greater in 3xTg-AD males than females (Genotype × Sex: *F*_1, 68_ = 8.921, *p* = .004, η2p = .116). No significant third order interaction was detected, but between-group comparisons with *t* tests revealed that CY-treated 3xTg-AD males had lower Aβ_42_ load than Veh-treated controls (*p* < .01). **e** BDNF mRNA levels were downregulated in 3xTg-AD mice of both sexes compared to controls. CY did not affect BDNF mRNA levels (normalized to β-actin) in 3xTg-AD mice. **f** 3xTg-AD mice treated with vehicle only had significantly higher *H2afy* mRNA expression compared to controls (*p* < .0001). CY treatment reversed the upregulated *H2afy* expression observed in vehicle-treated 3xTg-AD mice compared to WT mice (*p* = .046). No significant third order interaction was detected, but between-group comparisons with *t* tests revealed *H2afy* downregulation in CY-treated 3xTg-AD females. WT Veh males (*n* = 11), WT CY males (*n* = 11), 3xTg-AD Veh males (*n* = 9), 3xTg-AD CY males (*n* = 9), WT Veh females (*n* = 13), WT CY females (*n* = 14), 3xTg-AD Veh females (*n* = 10), 3xTg-AD CY females (*n* = 11). Overall group comparisons were carried out using three-way ANOVA (Genotype × Treatment × Sex), followed by post hoc *t* tests. Error bars = SEM, *p ≤ .05, **p < .01, ***p < .001. Abbreviations: Aβ, amyloid-beta; BDNF, brain-derived neurotrophic factor; mRNA, messenger RNA; pTau, phosphorylated tau; tTau, total tau; TBS, tris-buffered saline

Despite abolishing manifestations of systemic autoimmunity, CY treatment failed to normalize lower brain mass in ~ 7-month-old 3xTg-AD mice (Genotype: *F*_1, 120_ = 83.032, *p* < .001, η2p = .41, data not shown). Consistent with a growing consensus that neuronal damage in AD is triggered by soluble oligomers [45, 106], lighter brains in 3xTg-AD mice coincided with sex-dependent differences in TBS-soluble total tau and phospho-tau (Thr181) levels in the cortex (representative western blots are shown in Fig. 5a). Densitometric analysis revealed that 3xTg-AD females had elevated levels of TBS-soluble total tau in comparison to WT controls, but a similar elevation was not noted in 3xTg-AD males (Genotype × Sex: *F*_1, 81_ = 18.77, *p* < .001, η2p = .19, Fig. 5b). 3xTg-AD females also exhibited an increase in phospho-tau in comparison to all other groups (Genotype × Sex: *F*_1, 81_ = 16.354, *p* < .001, η2p = .18, Fig. 5c). Importantly, immunosuppression with CY had no appreciable effect on protein levels of TBS-soluble tau or phospho-tau species. In contrast to the findings with TBS-soluble tau species, sarkosyl-insoluble tau levels were not elevated in 3xTg-AD females or males compared to WT (data not shown).

Coinciding with the increase in soluble tau, 3xTg-AD mice also displayed elevated Aβ_42_ levels in TBS-soluble fractions of the cortex (Genotype: *F*_1, 68_ = 193.776, *p* < .001, η2p = .74, Fig. 5d). However, unlike tau, this increase in soluble human Aβ_42_ was greater in 3xTg-AD males than females (Genotype × Sex: *F*_1, 68_ = 8.921, *p* = .004, η2p = .116; 3xTg-AD males > 3xTg-AD females, *t*_28_ = 2.249, *p* = .033). Although significant Genotype by Sex by Treatment interaction was not detected with the present sample size, between-group comparisons with a *t* test revealed the most profound effect in 3xTg-AD males (CY-treated < Veh-treated, *t*_15_ = 3.325, *p* < .01).

Soluble phospho-tau [97] and Aβ species [39, 86] may exert their neurotoxic effects at least in part by downregulating BDNF expression, which is lower in the cortices of 3xTg-AD mice than in WT [56]. Given the essential role of BDNF downregulation in pre-clinical stages of AD [34, 87] and its links to anxiety-like behaviors [23], we examined BDNF mRNA levels to determine if its cortical expression is altered after generalized immunosuppression. However, CY failed to normalize BDNF expression in 3xTg-AD mice (Genotype: *F*_1, 80_ = 4.575, *p* = .035, η2p = .05, Fig. 5e).

Histone variants, which replace canonical histones in nucleosomes, were recently implicated in neural plasticity [66, 70, 123] and neurodegeneration [31, 49, 79]. We previously found that 3xTg-AD males, but not females, exhibit elevated expression of the histone variant macroH2A1 (mH2A1) compared to WT controls [56]. In the current study, 3xTg-AD mice exhibited increased *H2afy* expression (the mRNA for mH2A1 protein) in comparison to age-matched WT controls (Genotype: *F*_1, 74_ = 13.238, *p* < .001, η2p = .15). Interestingly, CY treatment normalized *H2afy* expression in the 3xTg-AD substrain (Genotype × Treatment: *F*_1, 74_ = 5.182, *p* = .026, η2p = .07, Fig. 5f). However, although no significant third order interaction was detected with the present sample size, this effect seemed to be driven by CY-treated 3xTg-AD females (CY-treated < Veh-treated, *t*_19_ = 3.148, *p* < .005). No significant between-group differences could be detected for *H2afy2* expression (data not shown), suggesting that CY exposure specifically modulates *H2afy* expression.

## Discussion

The current 3xTg-AD model exhibits an early anxiety-like phenotype that precedes the onset of fluctuating learning/memory deficits, as well as sex-specific markers of systemic autoimmunity and a delay in the progression of AD-like pathology [25, 50, 56, 69]. Here, we demonstrate that systemic immunosuppression failed to attenuate substrain dissimilarities in brain weight, soluble tau/phospho-tau, BDNF expression, or anxiety-related tasks. However, immunosuppressed 3xTg-AD males had improved hematocrit and lower Aβ load in the cerebral cortex. Compared to 3xTg-AD males, immunosuppressed females developed a distinct pattern of fur discoloration and showed downregulated expression of histone *H2afy* mRNA. Taken together, the results from our studies suggest that a sex-related autoimmune response in 3xTg-AD male mice increases soluble Aβ load in the cortex which coincides with altered transcriptional regulation of histone variant *H2afy* in females. Moreover, it appears that systemic autoimmunity does not fully account for the altered behavioral profile of 3xTg-AD mice. One may hypothesize that emerging autoimmunity is associated with either altered transcriptional regulation that has developed over time, a change in the mixture of background strains, or the pleiotropic effect of human transgenes acting on the immune system directly or via sex hormones. Since information pertaining to the status of the immune system in the original 3xTg-AD cohorts is lacking, the role of human transgenes in accounting for autoimmune manifestations could be addressed in the future by characterizing the immune status of PS1 and tau mice that show spleen enlargement [67]. Since human Aβ has recently been reported in the spleens of 15-month-old 3xTg-AD males [29] and blood of 5–9-month-old 3xTg-AD females [25], we may speculate that peripheral Aβ might further exacerbate peripheral immune responses.

Similar to many patients with mild cognitive impairment [68], enhanced emotionality and anxiety-like behaviors characterize the phenotype of 3xTg-AD mice from an early age and increase in severity over time [38, 41, 50, 69, 108]. Consistent with our work, enhanced emotionality in 6-month-old 3xTg-AD males coincides with an accumulation of soluble Aβ in the amygdala [33]. However, it has been also repeatedly reported that functional impairments in 3xTg-AD mice do not necessarily correlate with Aβ burden in the brain [4, 30, 53, 72, 85, 121]. In the current study, generalized immunosuppression did not abolish anxiety-related behaviors in 3xTg-AD males, but it reduced soluble Aβ in the cortex. This finding is particularly interesting in light of reports that Aβ itself may have innate immune functions like antimicrobial activity [63, 76] and that disruption of immune pathways attenuates Aβ burden [94]. Restoration of increased splenic Foxp3+ Tregs to basal levels has previously been shown to coincide with a reduction in the expression of Aβ in the hippocampus of 3xTg-AD males [29]. Using CY, we see similar lessening of Aβ burden in the cortex of 3xTg-AD males and females. Tregs play a major role in suppression of autoimmune pathology and are often poorly functioning in subjects with autoimmune disease [118]. It is therefore possible that the increase in the proportion of Tregs in 3xTg-AD mice is a protective reaction to enhanced autoimmune responses in these animals. Mitigating the influence of systemic Foxp3+ Treg-mediated immunosuppression on immunocytes may allow a re-balance of the immune response and reduced brain accumulation of Aβ [9]. One may speculate that the altered behavioral performance of 3xTg-AD mice reflects allostatic load due to autoimmune-mediated clearance of neurotoxic aggregates from the brain [105]. Females may not be able to readily mount such an immune response, rendering them vulnerable to plaque/tangle accumulation at older ages. Indeed, we found that 3xTg-AD females, but not males, exhibit an earlier rise in the amount of total and phosphorylated (Thr181) soluble tau in the cortex in comparison to WT controls. These findings complement recent immunohistochemical data documenting that 100% of 6-month-old 3xTg-AD females exhibit phospho-tau (Ser202/Thr205 and Ser422) in the hippocampus whereas male 3xTg-AD mice show considerable neuropathological variability [10]. This sex discrepancy in soluble total and phosphorylated tau may help to explain why accumulations of hyperphosphorylated tau tangles are observed in the brains of 12-month-old 3xTg-AD females [25] but not males [69].

Although the production of antibodies to Aβ and other antigens (nuclear and dsDNA) in 3xTg-AD males is consistent with clinical studies reporting autoantibodies in AD patients [51], their presence in WT females is an unexpected finding that requires further investigation. We previously noted that ~ 75% of aged WT males also showed varying degrees of ANA positivity [69]. These unexpected results in the WT mice of both sexes support the notion that the hybrid strain generated from 129 and C57BL/6 mice (ancestor to both the WT and 3xTg-AD strains) is predisposed to spontaneously develop autoimmune manifestations [18, 20]. However, why the insertion of AD-related genes accelerates the progression of autoimmune manifestations (in males in particular) remains to be determined. Similarly, more data are required to reveal the nature of the sex-specific patterns in depigmentation in 3xTg-AD mice exposed to CY. Hair graying, a typical sign of aging in mammals, has previously been linked to irreparable DNA damage that impairs the maintenance of melanocyte stem cells with age [52]. It remains to be determined if the graying in CY-treated 3xTg-AD mice reflects an increased accumulation of phosphoramide mustard (the cytotoxic metabolite of CY) leading to accelerated DNA damage and an early-aging phenotype.

Although the role of histone variants in the CNS is only beginning to be studied, existing data suggest that they are critical regulators of neural plasticity [70, 123]. The transcription of histone variants is highly responsive to environmental stimuli [123], including age-related regulation in the brain [70]. Histone macroH2A is a variant of the canonical histone H2A and is encoded by 2 genes that produce distinct proteins, *H2afy* (encodes mH2A1) and *H2afy2* (encodes mH2A2). We recently showed that *H2afy*, the mRNA for the histone variant mH2A1, is upregulated in the 3xTg-AD model, which is consistent with studies that demonstrate that *H2afy* is a marker of disease activity in neurodegenerative disorders [49]. The current study demonstrates that upregulated *H2afy* transcription can be modified by generalized immunosuppression. This suggests that upregulation of mH2A1 transcription may be driven by immune changes in AD. Since our data are limited to mRNA levels coding for mH2A1, we cannot draw conclusions about potential differences in mH2A1 histone variant incorporation into the chromatin of 3xTg-AD brains or its reversal with an immunosuppressive drug. However, transcriptional regulation of histone variants is related to altered histone variant incorporation [70, 123]. The effect of CY on histone variant expression was restricted to mH2A1, suggesting that mH2A1 is uniquely responsive to intervention and may play a role in AD pathology and chromatin dysregulation. However, a larger sample size is required to confirm the sex-specific difference in *H2afy* mRNA expression and to test its role in regulation of behavioral performance, fur appearance, or autoimmune markers in 3xTg-AD mice.

Taken together, the current study supports the hypothesis that development of systemic autoimmunity in 3xTg-AD mice modulates transcription of *H2afy* and soluble Aβ accumulation in a sex-specific manner. Since adult 3xTg-AD females show a more profound autoimmune profile than WT females, it is plausible that their intrauterine environment differentially affects brain development during embryogenesis of the offspring [28]. Alternatively, the organizational actions of sex steroid hormones during development are also linked to sex differences in Aβ accumulation in 3xTg-AD mice [21] and represent a future direction of inquiry.

The divergent effects of CY on certain pathological endpoints are likely due to its broad spectrum of activity as an alkylating agent that irreversibly interferes with the duplication of DNA in cells that divide frequently. While this mechanism of action makes it a potent immunosuppressant, CY may also cause beneficial immunomodulatory effects such as the induction of cytotoxic CD8^+^ T lymphocytes and CD4^+^ T_H_1 cells [107]. In keeping with previous observations [81], CY appeared to selectively promote an increase in the effector T cell population while decreasing the proportion of Tregs. This had the effect of normalizing 3xTg-AD T cell populations in relation to WT mice. The relative lack of effect on WT controls may seem counterintuitive at first, but is in line with evidence that autoimmune effector cells (including T cells and B cells) are uniquely sensitive to high-dose CY [16]. Although we did not count B cells in the current study, circulating autoantibody production was impaired after chronic exposure to CY. CY exhibits relative selectivity for T cells [1], but its known effects on B cells [59], dendritic cells [81], and macrophages [17] suggest some of the observed changes in 3xTg-AD mice may be due to differential effects on several immune responses. Although CY is also documented to affect non-immune cells within the hematologic, cardiovascular, gonadal, and gastrointestinal systems [32], the contribution of such systemic manifestations to AD-like pathology remains an ongoing topic of investigation [122]. While we cannot rule out the possibility that CY also directly affects soluble Aβ and expression of histone mH2A1 variant, only a small fraction of the alkylating metabolites of CY have been shown to cross the blood-brain barrier in mice [110]. Since CY and its metabolites may exert multisystem toxicity (immunosuppression is just one of its many effects), further experiments may benefit from more selective blockades of T cells, B cells, cytokines, or autoantibodies to elucidate terminal factors that account for changes in behavior and protein markers. Lastly, although epigenetic factors are known to be involved in establishing and maintaining immune responses [73], the current study does not reveal the mechanism(s) by which CY modulates epigenetic pathways.

Although our study points to early, sex-related immune activation as an important phenomenon, careful considerations need to be made when discussing its implications for clinical AD. At best, the 3xTg-AD model is representative of early-onset familial AD caused by genetic mutations in APP and PSEN1, which account for an estimated 1% or less of all AD-related dementia cases [5]. It remains unclear if familial forms of AD share a similar sex discrepancy with late-onset AD. Nevertheless, increasing evidence suggests that sex interacts with genetic factors to modify the risk for AD. For example, women carrying the ε4 allele of the apolipoprotein E gene (APOE4), the strongest genetic risk factor for late-onset AD, have a far more pronounced risk of developing AD than men carrying the allele [2, 120]. Moreover, several reports suggest that a maternal family history of AD confers higher risk for developing sporadic AD than paternal history or no family history [11, 48, 78]. The interactions between sex and genetic factors highlight the possibility that familial forms of AD may also be affected by sex differences. It also remains unclear to what extent, if any, brain-reactive autoantibodies and Tregs attenuate brain pathology by counteracting neuroinflammation in clinical AD [105]. Despite unknown mechanisms, the 3xTg-AD model may be a valuable in vivo model for studying interactions between autoimmunity and AD-like neurodegenerative brain disorders.

## Limitations

The main limitation of this study is the use of a mouse model that differs substantially in phenotype from its original description in 2003. Namely, the delay in AD-like neuropathology in recent cohorts of male mice and temporal disconnection between plaque/tangle formation and behavioral deficits calls into question the underlying assumptions of the 3xTg-AD model. Nevertheless, the early emergence of spontaneous systemic autoimmunity first detected in 3xTg-AD mice in 2013 suggests a potential mechanism that plays a role in regulating AD-like neurodegeneration, thus begging further investigation.

Our epigenetic data are limited to the measurement of mRNA levels. Therefore, we cannot draw conclusions about potential differences in mH2A1 histone variant incorporation into the chromatin of 3xTg-AD brains, or the mechanism by which its transcription is modulated by sustained immunosuppression. Along the same lines, cyclophosphamide affects a broad spectrum of cells and does not allow us to pinpoint which of T cells, B cells, cytokines, and/or autoantibodies constitute key factors in mediating its effects on multiple molecular and immunological dependent variables. Lastly, although this study reveals sex-specific effects of generalized immunosuppression at different system levels, it does not identify the origin of autoimmune phenomena in 3xTg-AD mice.

A behavioral experiment involving a 2 × 2 × 2 design (with Genotype, Sex, and Treatment as main factors) can be considered an overly ambitious endeavor. Indeed, it required the testing of three separate mouse cohorts to achieve a suitably large sample size (*N* > 100) in order to detect effects of medium size in three sets of variables. Given such a complex design, our project lasted almost 3 years and involved different groups of unbiased experimenters who performed behavioral experiments, which inherently generated variability among groups. Lastly, a behavioral battery followed by multiple comparisons increased the possibility of detecting significant *p* values by chance and of committing a type I error. We used MANOVA in preliminary data analysis and tempered our interpretations of significant task-specific differences in the “Discussion” section to minimize false inference and overstatement.

## Conclusions

The 3xTg-AD model is characterized by sex-related systemic autoimmunity, early anxiety-like behaviors, and transcriptional changes in epigenetic factors. We show that chronic immunosuppression with CY prevents hepatosplenomegaly and hypergammaglobulinemia and restores the phenotype of splenic T cells yet does not improve 3xTg-AD performance in anxiety-related tasks or increase brain mass or BDNF or lower phospho-tau levels. Sex-specific, reduced production of soluble Aβ, expression of histone mH2A1 variant, and fur graying suggest that chronic CY exposure has broad spectrum and sex-specific effects on molecular CNS markers and peripheral tissues. Collectively, our work suggests that systemic autoimmunity promotes specific prodromal markers of AD-like pathology and epigenetic markers of neurodegeneration, which jointly may contribute by yet unknown mechanisms to phenotypic alterations in the 3xTg-AD model.

## Supplementary Information

**Additional file 1: Supplemental Data.** Representative photos of femurs from 6-month old 3xTg-AD mice and WT controls treated with cyclophosphamide or vehicle.

## Data Availability

The datasets used and/or analyzed during the current study are available from the corresponding author on reasonable request.

## Abbreviations

3xTg-AD: Triple transgenic mouse model of Alzheimer’s disease
AD: Alzheimer’s disease
Aβ: Amyloid-beta
ANA: Anti-nuclear antibody
ANOVA: Analysis of variance
ANCOVA: Analysis of covariance
BDNF: Brain-derived neurotrophic factor
CY: Cyclophosphamide
dsDNA: Double-stranded DNA
ELISA: Enzyme-linked immunosorbent assay
FMO: Fluorescence-minus-one
FSC: Forward scatter
mH2A1: MacroH2A1
PBS: Phosphate-buffered saline
qRT-PCR: Quantitative reverse transcription polymerase chain reaction
SSC: Side scatter
pTau: Phosphorylated tau
tTau: Total tau
TBS: Tris-buffered saline
WT: Wild-type
