# Supplementary material for: Differential effects of chronic immunosuppression on behavioral, epigenetic, and Alzheimer’s disease-associated markers in 3xTg-AD mice
Source: Alzheimers Res Ther. 2021 Jan 20;13:30. doi: 10.1186/s13195-020-00745-9 (PMC7818784; doi:10.1186/s13195-020-00745-9)

Supplemental Data : *Femur of 6-month old 3xTg-AD and WT*

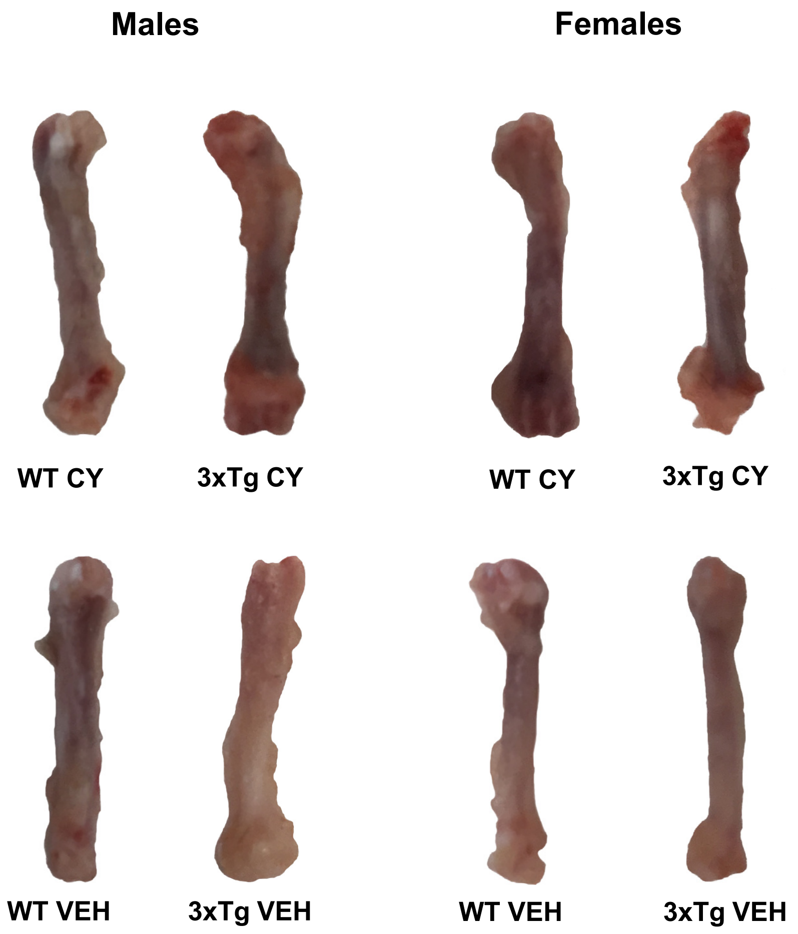

Supplement: Supplementary file 1 — Additional file 1: Supplemental Data. Representative photos of femurs from 6-month old 3xTg-AD mice and WT controls treated with cyclophosphamide or vehicle. [file 13195_2020_745_MOESM1_ESM.pdf]
